# Species delineation using Bayesian model-based assignment tests: a case study using Chinese toad-headed agamas (genus *Phrynocephalus*)

**DOI:** 10.1186/1471-2148-10-197

**Published:** 2010-06-25

**Authors:** Daniel WA Noble, Yin Qi, Jinzhong Fu

**Affiliations:** 1 Department of Integrative Biology, University of Guelph, Guelph, Ontario N1G 2W1, Canada; 2 Chengdu Institute of Biology, Chinese Academy of Sciences, Chengdu, Sichuan 610041, China

## Abstract

**Background:**

Species are fundamental units in biology, yet much debate exists surrounding how we should delineate species in nature. Species discovery now requires the use of separate, corroborating datasets to quantify independently evolving lineages and test species criteria. However, the complexity of the speciation process has ushered in a need to infuse studies with new tools capable of aiding in species delineation. We suggest that model-based assignment tests are one such tool. This method circumvents constraints with traditional population genetic analyses and provides a novel means of describing cryptic and complex diversity in natural systems. Using toad-headed agamas of the *Phrynocephalus vlangalii *complex as a case study, we apply model-based assignment tests to microsatellite DNA data to test whether *P. putjatia*, a controversial species that closely resembles *P. vlangalii *morphologically, represents a valid species. Mitochondrial DNA and geographic data are also included to corroborate the assignment test results.

**Results:**

Assignment tests revealed two distinct nuclear DNA clusters with 95% (230/243) of the individuals being assigned to one of the clusters with > 90% probability. The nuclear genomes of the two clusters remained distinct in sympatry, particularly at three syntopic sites, suggesting the existence of reproductive isolation between the identified clusters. In addition, a mitochondrial ND2 gene tree revealed two deeply diverged clades, which were largely congruent with the two nuclear DNA clusters, with a few exceptions. Historical mitochondrial introgression events between the two groups might explain the disagreement between the mitochondrial and nuclear DNA data. The nuclear DNA clusters and mitochondrial clades corresponded nicely to the hypothesized distributions of *P. vlangalii *and *P. putjatia*.

**Conclusions:**

These results demonstrate that assignment tests based on microsatellite DNA data can be powerful tools for distinguishing closely related species and support the validity of *P. putjatia*. Assignment tests have the potential to play a significant role in elucidating biodiversity in the era of DNA data. Nonetheless, important limitations do exist and multiple independent datasets should be used to corroborate results from assignment programs.

## Background

Species are fundamental units in biology, yet conceptually defining them has been a difficult task owing to the complexity and continuity of the speciation process itself and the diversity of reproductive modes among organisms. Attempts to resolve this debate have lead to the proliferation of different species concepts, most of which embody important aspects of the speciation process but lack a general applicability necessary for characterizing all species diversity. Over the last few decades the debate surrounding what exactly species are seems to be moving towards a consensus. Synthesizing similarities among different species concepts, both Mayden [1] and de Queiroz [2] recognized that most species concepts are not primary definitions describing the necessary traits of a species, but rather, are secondary definitions or species criteria. In general, species are separately evolving population level lineages, which may acquire unique traits as they evolve [1-3]. Under this framework, the operational criteria of most species concepts are used to quantitatively assess the degree to which lineages have diverged and are used as evidence to describe species. With an emerging consensus on the definitive properties of a species, there has been a shift to focusing on ways to delimit species in nature [3-8].

Methods of delineating species are numerous and can be grouped into what have been deemed 'non tree-based' and 'tree-based' methods [5]. Tree-based methods vary, but all make use of either morphological or molecular data to estimate phylogenetic relationships of individuals that share a common evolutionary history [5]. These methods rely heavily on the monophyly criterion to make species designations, which assumes that all populations of the same species share a most recent common ancestor with each other (but see [9]). In contrast, non-tree based methods have traditionally relied on the concept of reproductive isolation between species. Although such isolation can be directly tested occasionally, most methods infer reproductive isolation by indirectly estimating gene flow within and between hypothesized species.

The reproductive isolation criterion recognizes that reduced gene exchange plays a pivotal role characterizing species boundaries [10,11] and is widely accepted among evolutionary biologists as being an important process leading to the divergence of sexually reproducing species [12]. Gene flow has traditionally been characterized using protein based allozyme markers to assess the level of genetic divergence between pre-defined populations [13-16]. Fixed allelic differences at multiple loci were considered strong evidence for reproductive isolation. Although effective, this requires the sacrifice of organisms, which for many groups is no longer feasible. Moreover, allozyme markers are often too conservative to delineate closely related species. Molecular markers such as short tandem repeats (e.g. microsatellite DNA) have gained major popularity among both ecologists and evolutionary biologists because genomic DNA can be readily extracted from a small amount of tissue [17,18]. In addition, these markers are readily available, relatively inexpensive and amplify across closely related species [17,18]. Important innovations in population genetic analysis and greater computational power have allowed researchers to make use of model-based assignment tests [17,19], which may provide powerful tools for inferring reproductive isolation and species boundaries.

Assignment tests have become popular tools for assessing a multitude of questions of both applied and theoretical importance [19]. These tests make use of Bayesian or likelihood statistics to cluster individuals based on linkage disequilibrium in a sample of individuals from distinct populations [19]. Linkage disequilibrium occurs when a population has a non-random association between genotypes at multiple loci, and admixture between two distinct populations is one common cause of linkage disequilibrium [10,20,21]. Assignment tests attempt to decompose such mixture by creating clusters of individuals within which linkage disequilibrium is minimized [19]. These tests can simultaneously define naturally occurring clusters of individuals based on their multi-locus genotypes and assign individuals to clusters. Traditionally, these tests have been used in fisheries science to identify the origin of a particular fish, however, they have since diversified to include the identification of illegal harvests [22], establishing the origins of bioinfestations [23], identifying hybrids [24,25], assessing population structure [26], and determining the number of migrants from other populations [27]. Assignment tests provide important advantages over traditional population genetic analyses because: 1) they do not require large sample sizes; 2) there is no need to establish populations *a priori *[19,28]; 3) they treat individuals as the units of analysis, therefore increasing power to detect subtle biological processes [28]; 4) they allow for the detection of admixed or hybrid individuals [19,28] and 5) they allow for the incorporation of geographic information [29]. Manel et al. provides an excellent review of assignment tests [19]. To date few studies have directly used assignment tests for species delineation and none have formally discussed their general potential as a tool for species delineation, despite their successes [30,31]. Assignment methods in conjunction with neutral, co-dominant markers, such as microsatellite DNA repeats, may provide researchers with a powerful means of establishing species boundaries because they can be easily applied and address numerous species criteria, such as the genotypic clustering criterion proposed by Mallet [32] and the reproductive isolation criterion [33]. However, case studies are necessary to determine their general applicability.

**Figure 1:**
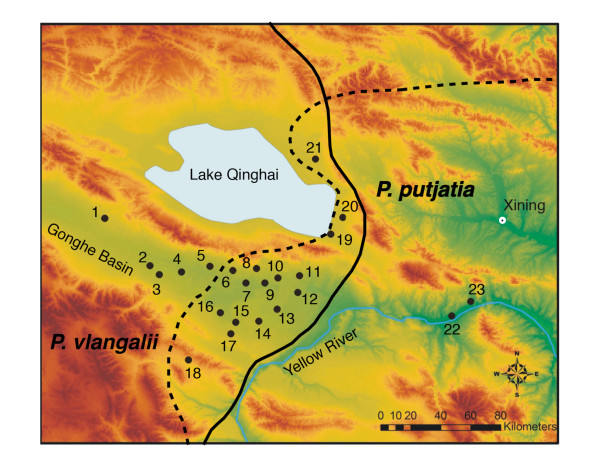
**Map of collecting sites (1-23) in Gonghe Basin and Lake Qinghai area of China, where *Phrynocephalus vlangalii *(solid line) and a proposed species, *Phrynocephalus putjatia *(dashed line), are sympatric**. Samples from site 1, 18 and 21 were collected in 2007 and all others were collected in 2008.

Toad-headed lizards of the *P. vlangalii *complex provide an excellent model system to test species boundaries using such approaches because they are widespread, numerous primers exist that cross amplify microsatellite DNA from closely related species, and they have a well established mtDNA phylogeny and have received substantial taxonomic attention. The high morphological variability within *P. vlangalii *has generated intense debate over species designations providing an interesting and difficult case to test species level boundaries. One specific case occurs between *P. vlangalii *and *P. putjatia*. Bedriaga assigned populations of '*P. vlangalii*' southeast of Lake Qinghai (= Lake Kukunor) to *P. putjatia *based on morphological differences [34]. However, the status of *P. putjatia *has been contentious; while Wang *et al*. considered it a valid species based on morphological and chromosomal differences [35], most authors regard it as a synonym of *P. vlangalii *[36-38]. Recently, Jin *et al*. revealed two deeply diverged mitochondrial DNA (mtDNA) clades situated in the eastern and western areas of '*P. vlangalii's' *distribution [39], which appears to correspond to *P. putjatia *and *P. vlangalii*, respectively. Molecular clock estimates suggest that the initial divergence between these two clades took place approximately 6.43-4.75 million years ago [39]. Surrounding Lake Qinghai these two proposed species overlap, which provides an excellent opportunity to test the species hypotheses (Figure 1).

We apply Bayesian assignment methods to resolve the relationships between *P. vlangalii *and *P. putjatia*. A large number of individuals from the presumptive distribution of both species, including the sympatric zones, are examined using eight microsatellite DNA markers and a mitochondrial gene (ND2). If *P. putjatia *is a synonym of *P. vlangalii *(*i.e*. one species hypothesis), we would predict a single nuclear DNA cluster containing many individuals from both mtDNA clades. In contrast, if *P. putjatia *is a valid species (*i.e*. two species hypothesis), we would predict that two genotypic clusters will exist, and they will correspond to the previously identified mtDNA clades [39]. In addition, nuclear genotypes of both clusters should persist in the sympatric zone.

## Results

### Microsatellite DNA

We examined 243 individuals with eight microsatellite DNA loci. Allelic diversity ranged from 36 (PVMS38) to 71 (Phry75) alleles per locus with an average of 47.5 alleles/locus. MICRO-CHECKER version 1.0 [40] did not detect evidence for large allele drop out or scoring errors, however, it did detect the potential for null alleles at Phry79, Phry75 and PVMS35.

**Figure 2:**
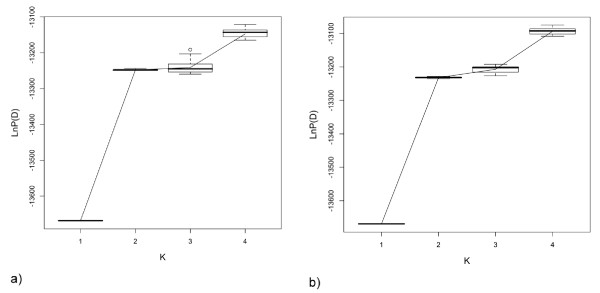
**Avereage likelihood values from STRUCTURE**. Boxplots of the 13 highest likelihood values of 100 independent runs for K = 1-4 from STUCTURE: a) admixture model; b) no admixture model.

**Figure 3:**
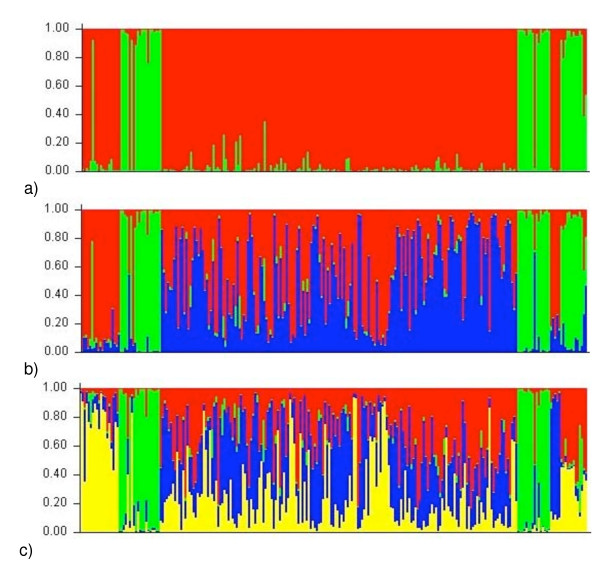
**Individual assignment probabilities from STRUCTURE for: a) K = 2, b) K = 3 and c) K = 4**. Each vertical line represents one individual and each colour represents a single cluster. The vertical height of each colour represents the probability of being with that colour.

We used the program STRUCTURE version 2.2 [41] to detect the number of naturally occurring clusters (K) within the examined individuals. Average LnP(D) values from STRUCTURE increased by 3.3% from K = 1 to K = 2, 0.19% from K = 2 to K = 3 and 0.87% from K = 3 to K = 4 (Figure 2). The large jump in LnP(D) values between K = 1 and K = 2 followed by small changes between K = 2 and K = 3 suggest that K = 2 is adequate to explain the data and is also the most parsimonious. In addition, individual assignment probabilities declined as K increased to 3 and 4 (Figure 3). When K = 2, the two clusters were distinct (Figure 3a) with 95% (230/243) of the individuals assigned with > 90% probability to one of the clusters. The two clusters had an average F _ST _= 0.0524 and 5% of individuals showed evidence of an 'admixed' nuclear genome. When K = 3 or 4, the clusters became indistinct with the majority of individuals having an assignment probability of less than 80% (K = 3, 52% and K = 4, 62%; Figure 3b,c). Due to the large fragment size and high allelic diversity at locus Phry75, we removed it from the analysis to determine whether it influenced assignment probabilities. Re-running the analysis without this locus caused little change in assignment probabilities.

**Figure 4:**
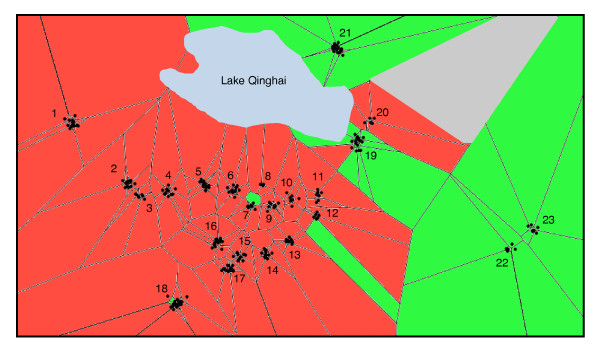
**Spatial clustering based on individual assignment probabilities from TESS (version 2.1)**. Population coordinates were permuated using a standard deviation of 0.015 to more accurately depict individuals. Each dot represents a single individual. Red corrosponds to the *Phyrnocephalus vlangalii *cluster and green corresponds to the *Phrynocephalus putjatia *cluster. Grey polygon represents a lack of information

**Figure 5:**
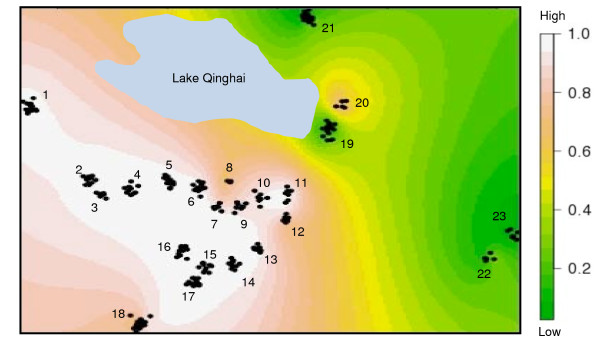
**Interpolated admixture levels in the red cluster (*P. vlangalii*)**. White colour represents individuals with a high proportion of their genome assigned to the red cluster while dark green represents individuals with a low proportion of their genome being assigned to the red cluster. Admixture values are computed for each of the cells within the geographic area of interest.

We used the program TESS version 2.1 [29] to incorporate geographic information into the analysis. Assignment probabilities from TESS were nearly identical to the STRUCTURE results. TESS identified a red cluster (*P. vlangalii*) that was situated south of Lake Qinghai and a green cluster (*P. putjatia) *that was situated east of Lake Qinghai (Figure 4), which correspond to the geographic locations of the two deeply diverged mtDNA lineages described by Jin *et al*. [39] and the hypothesized species ranges [35,39]. The two clusters displayed a large overlap zone (sympatry) and members of the two clusters also co-existed at sites 18, 19 and 21 (syntopy). Furthermore, individuals southwest of Lake Qinghai had a large proportion of their genome assigned to the red cluster as indicated from the high level of admixture among individuals in this region with the red cluster (*P. vlangalii)*, while individuals in the east showed little admixture with the red cluster (Figure 5). An opposite pattern was found for the green cluster (diagram not shown). It would have been desirable to obtain more samples further east of Lake Qinghai to compare more thoroughly the extent to which the nuclear DNA showed geographic congruence with the hypothesized species distributions. However, this was not possible because the areas bordering the Yellow River are characterized by agriculture, providing un-suitable habitat for these lizards.

There were few individuals with admixed nuclear genomes. Thirteen individuals (5%) had less than 90% probability and seven had less than 80% probability of being assigned to a cluster. Within the three syntopic sites (18, 19 and 21), only one individual out of 54 individuals examined had an admixed genome. From site 18, all 19 individuals received >90% probability of being assigned to one cluster; 18 were assigned to the *P. vlangalii *cluster and one was assigned to the *P*. *putjatia *cluster. Site 19 has a similar situation: one was assigned to the *P. vlangalii *cluster and 14 were assigned to the *P*. *putjatia *cluster, and all received >90% probability. The only individual with an admixed genome was from site 21 and it had a probability of 0.729 of belonging to the *P*. *putjatia *cluster. Two of the remaining individuals were assigned to the *P. vlangalii *cluster and 18 to the *P*. *putjatia *cluster.

### Mitochondrial DNA

A total of 146 individuals were successfully sequenced for an 850 base pair DNA fragment of the mitochondrial ND2 gene. Additionally, 19 sequences were obtained from Jin *et al*. [39], which had an approximately 350 base pair overlap with our data. Sequences for *Phrynocephalus theobaldi*, *P. zetangensis*, *P. axillaris *and *P. versicolor*, were obtained from GenBank and used as outgroup taxa. The alignment was straightforward and produced no gaps. Overall, 74 unique haplotypes were identified including 70 ingroup members and four outgroup members.

**Figure 6:**
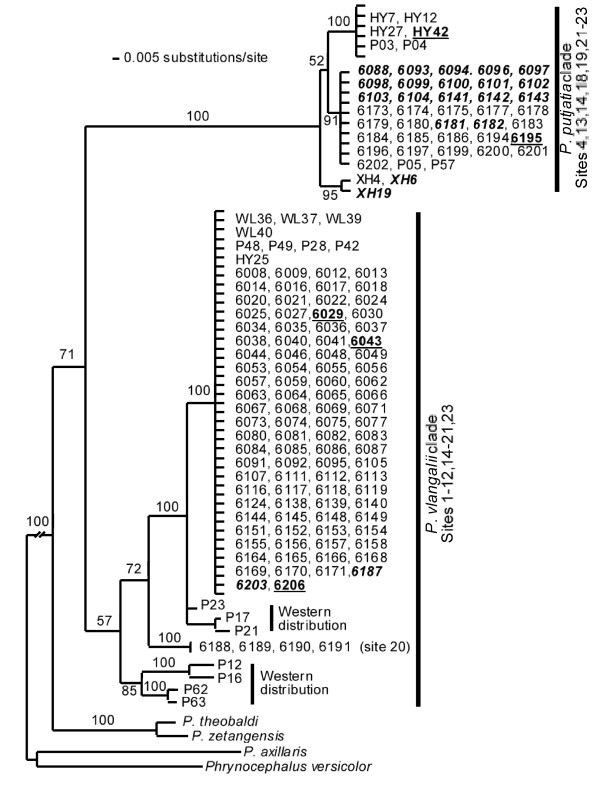
**Bayesian phylogenetic tree for 146 individuals using a 850 base pair fragment of the mitochondrial ND2 gene**. A total of 19 sequences of the ND2 fragment from Jin *et al*. [39] were also included in this analysis and are labeled as P#. Only posterior probabilities associated with the basal nodes are included and tip nodes with low support were collapsed to simplify the tree. Underlined and bolded individuals have admixed nuclear genomes and italicized and bolded individuals have a pure nuclear genome from the opposite species.

A Bayesian analysis with MrBayes version 3.2 [42] was conducted to reconstruct the historical relationships among the haplotypes. A GTR + G model was selected to be the best-fit model based on Akaike's Information Criteria. The Bayesian tree along with posterior probabilities is shown in Figure 6. Our tree topology and population designation were nearly identical to those of Jin *et al*. [39]. Two clades were clearly identified. A *P*. *vlangalii *clade was primarily situated in the western part of the Gonghe Basin south of Lake Qinghai, except for site 20. Members of this clade occurred in sites 1-12, 14-21 and 23 (Figure 1 &6). A *P*. *putjatia *clade was primarily found in the Gonghe Basin east of Lake Qinghai, including sites 4, 13, 14, 18, 19 and 21-23. (Figure 1 &6).

### Congruence between Microsatellite and Mitochondrial DNA Data

Individual membership to each of the two nuclear DNA clusters was largely congruent with the mitochondrial DNA clades, with a few exceptions. The complete genotypes of 146 individuals with both mitochondrial and nuclear gene data are provided in Table 1. All individuals from site 13 had a mtDNA haplotype from *P. putjatia *but a nuclear genome entirely composed of *P. vlangalii *(*vv *_*p*_). In contrast, one individual from site 19 and one individual from site 23 had a *P. vlangalii *mtDNA haplotype but a *P. putjatia *nuclear genome (*pp *_*v*_). Individuals with mixed mitochondrial and nuclear genomes also occurred at sites 4, 14 and 18 (Table 1). Overall, 82% of all individuals contained pure genomes (*pp *_*p *_or *vv *_*v*_), 15% of all individuals had pure nuclear genomes and the opposite mitochondrial genomes and 3% of all individuals contained an admixed nuclear genome with a *P*. *putjatia *or *P*. *vlangalii *mtDNA haplotype.

**Table 1:** Percentage of mitochondrial (mtDNA) and nuclear genotypes at each sampling site

Population	***vv***_***v***_	***vv***_***p***_	***pp***_***p***_	***pp***_***v***_	**AD**_***v***_	**AD**_***p***_	N
1	100%	-	-	-	-	-	4
2	100%	-	-	-	-	-	10
3	100%	-	-	-	-	-	4
4	62.5%	37.5%%-	-	-	-	-	8
5	100%	-	-	-	-	-	4
6	100%	-	-	-	-	-	6
7	100%	-	-	-	-	-	7
8	100%	-	-	-	-	-	3
9	80%	-	-	-	20%	-	5
10	100%	-	-	-	-	-	6
11	100%	-	-	-	-	-	5
12	66.7%	-	-	-	33.3%%	-	3
13	-	100%	-	-	-	-	9
14	62.5%	37.5%%	-	-	-	-	8
15	100%	-	-	-	-	-	9
16	100%	-	-	-	-	-	10
17	100%	-	-	-	-	-	9
18	-	66.7%	33.3%	-	-	-	3
19	-	14.3%	78.6%	7.1%	-	-	14
20	100%	-	-	-	-	-	4
21	20%	-	60%	-	-	20%	5
22	-	-	83.3%	-	-	16.6%	6
23	-	-	50%	25%	25%	-	4

*vv*_*v *_represents individuals with a nuclear genome composition and mtDNA haplotype from *Phrynocephalus vlangalii*. *vv*_*p *_represents individuals with a nuclear genome composition from *Phrynocephalus vlangalii *and a mtDNA haplotype from *P. putjatia*. "AD" refers to admixed nuclear genomes. The sample size represents individuals that both mitochondrial and nuclear genotypes are known.

## Discussion

### Validity of *Phrynocephalus putjatia*

We reject the one species hypothesis (*i.e*. all populations are *P. vlangalii*). Three lines of evidence support this conclusion: 1) assignment tests distinguish two naturally occurring distinct nuclear DNA clusters, 2) the existence of few intermediates in the sympatric zone indicates that members of the two genetic clusters rarely hybridize, and 3) the clusters largely correspond to the two deeply diverged mtDNA clades and the geographic locations of the two proposed species. This evidence suggests established reproductive isolation between the clusters, and therefore, they should be recognized as distinct species, i.e. *P. vlangalii *and *P. putjatia*, according to the biological species concept (criterion).

The largest change in LnP(D) values occurred between K = 1 and K = 2 suggesting that two very distinct gene pools exist in the dataset. In addition, individuals could be assigned to one of the two identified clusters with greater than 90% probability further supporting discrete differences at the nuclear genetic level. Furthermore, the clusters exhibited geographic cohesiveness (Figure 4 &5). Jin *et al*. suggested that *P. putjatia *diverged from all other populations of *P. vlangalii *during the Late Miocene when global temperatures decreased and the climate became more cool and arid [39]. Ancestral populations isolated during this time would have diverged substantially from other populations.

Although two distinct clusters were identified, the F _ST _value between these clusters was low (average F _ST _= 0.0524). Genetic divergence among species is known to be extremely variable, with F _ST _estimates ranging from 0.05 to over 0.77 based on allozyme markers [43]. One reason for the low genetic differentiation in our case is likely due to the high mutation rates among the loci used in this study. Loci with high mutation rates likely experience high levels of homoplasy decreasing the differentiation between groups and leading to low F _ST _values [44]. This is plausible given the high allelic diversity (47.5 alleles/locus) we observed. O'Reilly *et al*. showed that microsatellite DNA markers with high polymorphism caused a decline in F _ST _estimates between Walleye Pollock (*Theragra chalcogramma*) populations [44]. Simulation studies also suggest that increased mutation rates significantly decrease estimates of F _ST _[45,46] and Hedrick also analytically demonstrated that when the number of alleles is high, F _ST _is necessarily low [47]. Other explanations such as historical introgression through hybridization and the small sample sizes of *P. putjatia *are also possible. Greater sampling effort south of the Yellow river will be necessary to differentiate between these explanations.

The observed allelic diversity in this study (47.5 alleles/locus) was greater than that found by Wang *et al*., who found an average of 32.5 alleles per locus [48]. The greater average number of alleles per locus in this study is likely due to the different loci used and differences in microsatellite scoring between studies. Wang *et al*. found that populations from sites 1 and 18 had the greatest number of alleles on average than any other populations (23.8 and 16.5, respectively) [48]. Since this study had much more extensive sampling in this area it is not surprising that a greater diversity of alleles was found. Wang *et al*. speculated that the high genetic diversity in this group is likely due to the age of the lineage, the high population density of these lizards, isolation with migration and the lack of bottleneck events [48].

The maintenance of *P. vlangalii *and *P. putjatia *nuclear genomes in sympatry suggests that intrinsic barriers to gene flow exist, such as pre- or post-mating isolation mechanisms, preventing inter-breeding between these two species. The majority of our sampling sites are from the overlap zone between the hypothesized ranges of the two species (Figure 1). Within this broad sympatric zone, individuals with admixed nuclear genomes are few, only approximately 5%. Some sites (i.e. 19 and 20) are in close geographic proximity (< 5 km) without any obvious physical barriers, and yet, the genetic makeup of the two populations is different (Figure 1, Table 1). Furthermore, the two species are syntopic at sites 18, 19 and 21, and only one hybrid (out of 54 individuals) with an admixed nuclear genome was detected. If these were not two species one would expect most of the individuals examined to have admixed or hybrid genomes as a consequence of random mating between the two genotypes. Nevertheless, the presence of individuals with admixed nuclear genomes suggests recent hybridization events between *P. vlangalii *and *P. putjatia *and a lack of complete reproductive isolation. Furthermore, 15% of the individuals had disparate mtDNA and nuclear DNA suggesting the occurrence of historical introgressive hybridization events between *P. vlangalii *and *P. putjatia *in the Gonghe basin and east of Lake Qinghai (Table 1). The genus *Phrynocephalus *is known for its complex social structure and mating behaviours, which may play an important role in mate choice and intrasexual communication. To date, the ecology and behaviour of these species remain poorly understood, and future work addressing the mating behaviour of these lizards will help elucidate potential isolation mechanisms between these species.

Sites 4, 13, 14, 18 and 19 contained individuals with mtDNA haplotypes from *one species *but the nuclear genome entirely from the other species. Jin *et al*. provided evidence that *P. vlangalii *from the Qiadam Basin exhibited a rapid eastward range expansion as the Yellow River drained the paleao-Lake Gonghe, approximately 0.15 million years ago [39]. Under this scenario, *P. vlangalii *would have come into secondary contact with *P. putjatia *populations isolated during this time. Hybridization would have resulted in introgression of mtDNA haplotypes from both of these species. Continual backcrossing with one parental species would have "diluted" any evidence of hybridization in their nuclear genomes, while the introgressed mitochondrial genome remained in place. Indeed, historical hybridization and introgression of mtDNA haplotypes has been reported in a number of studies ranging from amphibians to mammals [49-51]. For example, Melo-Ferreira *et al*. reported extensive introgression in the Iberian Hares from Spain with 32% of *Lepus granatensis *containing an mtDNA lineage from *L. timidus *[49].

Our genetic clustering did not match the morphological diagnosis provided by Wang *et al*. [35], although the distribution of the genetic clusters was largely congruent with their proposed species ranges. Wang *et al*. suggested that *P. putjatia *has a tail length larger than its snout-vent length and has more than 100 rows of back scales, while *P. vlangalii *has a relatively short tail and fewer than 100 rows of back scales [35]. Approximately half of *P. putjatia *identified in this study did not possess these "diagnostic" characters. This is not particularly surprising given that the genus *Phyrnocephalus *is notorious for exhibiting large intraspecific morphological variation but lack of consistent interspecific differences [38,52]. Such phenotypic diversity has made it particularly difficult to delineate species and morphology-based taxonomy is very confusing in this genus. For example, more than ten names have been applied to *P. vlangalii *historically [38]. Rather than searching for a few diagnostic characters, a multivariate analysis, such as principle component analysis, may be more appropriate to dissect the morphological differences between *P. putjatia *and *P. vlangalii*. It is important to note that a lack of congruence between morphology and other tools (e.g. genetic) for diagnosing species should not necessarily be regarded as evidence against the validity of two species [8]. Traditional morphology-based taxonomy, is only one way to describe "life's diversity" and species hypotheses developed based on morphology alone should be tested using different approaches with a variety of data sets [8]. Wang *et al*. also described chromosomal differences between the two species; while *P. vlangalii *has ZW sex chromosomes, *P. putjatia *lacks identifiable sex chromosomes [35]. Unfortunately, we do not have chromosome data for specimens examined in this study. Nevertheless, the chromosome difference could potentially provide a reproductive isolation mechanism.

### Assignment Tests as a Tool for Species Delineation

The idea to treat species as cohesive genotypic clusters, with few intermediates, was first formulized by Mallet [32] and has since been adopted by two studies to clarify taxonomic issues between proposed species [30,31]. Drummond & Hamilton applied assignment tests in a sympatric zone between two varieties (*i.e*. sub-species) of *Lupinus microcarpus *[31]. They found strong evidence to suggest two distinct clusters corresponding to *L. microcarpus horizontalis *and *L. microcarpus densiflorus*, with the largest difference in likelihood values between K = 1 and K = 2. They concluded that the two varieties should be recognized as separate species because they maintain their distinctiveness in sympatry and show low levels of admixture (only 2.1% of individuals showed evidence of an admixed genome). Maingon *et al*. also used assignment tests to discern between sandfly (*Lutzomyia longipalpis*) populations exhibiting differences in pheromone composition [30]. They too found strong evidence that sandflies with different pheromone compounds (9 MGB and cembrene) are distinct species, with < 10% of all individuals being incorrectly assigned. These and the present study reveal the power of assignment tests in resolving difficult taxonomic issues.

Assignment tests will be most powerful where two species are sympatric, particularly in syntopic populations, because reproductive isolation can be more rigorously addressed in these situations. In sympatry, distinct genotypic clusters are most likely the product of intrinsic reproductive isolation between the clusters, and these clusters should merit species status according to the biological species criterion. In allopatry, however, isolation by distance and geography are known to cause differentiation between populations within species and can confound interpretations of species diversity because genetic clusters arising from these processes may be mistaken as separate species. Under these circumstances, multiple molecular and non-molecular tools should be used concurrently to assess evidence of species level designations.

A number of different molecular markers can be used in assignment programs, providing researchers with a diversity of tools for addressing species boundaries. Microsatellite DNA markers are probably the most commonly used markers for such analyses. Because of their high allelic diversity, they are capable of detecting subtle population genetic structure [17] and will be particularly useful where species have diverged little genetically. The use of microsatellite DNA will likely only apply to closely related species for which markers have been successfully characterized and cross amplify [17]. Other co-dominant markers, such as single nucleotide polymorphisms (SNPs), can also be used in assignment tests providing that the loci are independent and not in linkage disequilibrium within populations [41]. With the advent of new molecular techniques [53,54], it is now feasible to use hundreds of independent loci throughout the genome making the use of these methods more robust.

An important assumption of assignment test models is that populations are in Hardy-Weinberg and linkage equilibrium or nearly so [29,55,56]. These conditions are not satisfied by most empirical studies and greater effort should be put towards understanding how violations of such assumptions affect conclusions drawn from assignment test analyses.

## Conclusions

The results from the assignment tests and phylogenetic analyses support the validity of *P. putjatia *as being a distinct species. There are two distinct genetic clusters in sympatry and the clusters largely correspond to the hypothesized distributions of *P. vlangalii *and *P. putjatia *and two deeply diverged mtDNA lineages. However, there is evidence for both contemporary and historical hybridization between *P. vlangalii *and *P. putjatia*. The use of both nuclear and mitochondrial DNA markers will help researchers elucidate these processes in nature and more accurately depict the historical events that have taken place between taxa. Future studies should strive to include both nuclear and mitochondrial DNA information, and the use of assignment tests will facilitate this revolution.

Species delineation has always been an important scientific endeavor. The characterization of independently evolving lineages is necessary for addressing fundamental theories in ecology and evolution, while also guiding our efforts in conserving biodiversity. An evidence-based approach to species discovery requires the recruitment of a wide array of tools to effectively discriminate between competing species hypotheses. Assignment methods using microsatellite DNA markers provide a novel tool to test species hypotheses and will be especially beneficial when examining recently diverged species and in groups where collecting a large number of individuals from a single location is difficult.

## Methods

### Sample Collection and Location

A total of 243 individuals were examined in this study. Among them, 189 samples were collected in 2008; three to fifteen individuals were collected at 20 different sites along two transects (east to west and north to south) where the two previously identified mtDNA clades are known to persist in sympatry [39]. These two clades provide a unique opportunity to make use of assignment tests in assessing the reproductive isolation between these groups because: 1) they are geographically close together, reducing the possibility that genetic divergence is due to isolation by distance and 2) they are found in a small flat area, reducing the possibility that genetic divergence is a result of a topologically complex landscape. Specimens were euthanized by an intracardial injection of sodium pentobarbital. Liver and heart muscle were excised and stored in 95% ethanol and all voucher specimens were preserved in the Chengdu Institute of Biology. An additional 54 samples from three sites of the same area, which were collected in 2007 and used in a previous study [48] were also included in this study. Toe clippings were taken from all samples and no vouchers were collected in 2007. We assumed that the temporal genetic variation between the two years was minimal. A full list of samples and their collecting locations is provided in Table 2 and depicted in Figure 1.

**Table 2:** Sampling locality information, specimen numbers and sample sizes

Site	Catalogue Numbers	Latitude	Longitude	Sample Size
1	None (WL)	N 36.65243°	E 99.37298°	15
2	CIB 06148-6162	N 36.36917°	E 99.64797°	15
3	CIB 06163-6168	N 36.31429°	E 99.70199°	6
4	CIB 06135-6145	N 36.32911°	E 99.83822°	11
5	CIB 06117-6132	N 36.36355°	E 100.00814°	15
6	CIB 06105-6116	N 36.34046°	E 100.14559°	12
7	CIB 06033-6041	N 36.26327°	E 100.22209°	9
8	CIB 06169-6171	N 36.35032°	E 100.28450°	3
9	CIB 06023-6030	N 36.26527°	E 100.33526°	8
10	CIB 06016-6022	N 36.29575°	E 100.41498°	7
11	CIB 06008-6015	N 36.30566°	E 100.54564°	7
12	CIB 06042-6046	N 36.20682°	E 100.53485°	5
13	CIB 06096-6104	N 36.10402°	E 100.40946°	9
14	CIB 06086-6095	N 36.03330°	E 100.30091°	10
15	CIB 06062-6071	N 36.02903°	E 100.16232°	10
16	CIB 06072-6085	N 36.08624°	E 100.06871°	14
17	CIB 06048-6061	N 35.96003°	E 100.13024°	14
18	None (XH)	N 35.80006°	E 099.87933°	19
19	CIB 06173-6187	N 36.55759°	E 100.73343°	15
20	CIB 06188-6192	N 36.65764°	E 100.80575°	5
21	None (HY)	N 37.01030°	E 100.63975°	20
22	CIB 06194-6200	N 36.15355°	E 101.57399°	7
23	CIB 06201-6207	N 36.06605°	E 101.45580°	7

Site labels correspond to those on Figure 1.

### DNA Extractions and Microsatellite DNA Amplification

Genomic DNA was extracted from liver, muscle or toe clippings. Minced tissue was incubated overnight at 55°C in a solution containing 75 ul of 10% SDS (Sodium Dodecoly Sulfate), 600 ul of STE buffer and 17.5 ul of proteinase K (20 mg/mL). Samples were extracted twice with phenol: chloroform: isoamyl alcohol (25:24:1) and once with chloroform: isoamyl alcohol (25:1). Genomic DNA was precipitated with 3 M NaAc (pH 5.2) and -20°C 95% ethanol, washed with 70% ethanol twice and re-suspended in PCR grade water (Sigma) overnight.

We used eight microsatellite DNA loci (Phr27, Phr63, Phr75, Phr79, PVMS12, PVMS18, PVMS35, and PVMS38) for which primers were developed [57,58]. These loci have previously been used in population genetic studies of *P. vlangalii *[48] and *P. przewalskii *[59], and were shown to be in linkage equilibrium. These loci also had a close match between their expected and observed heterozygosity, which is indicative of a low occurrence of null alleles. PCR amplification was performed in 10 ul reaction volumes containing 0.5 ul of extracted DNA, 0.1 ul *Taq *polymerase (Takara; rtaq), 0.6 ul of Mg ^2 + ^(25 mM), 1.0 ul of 10× universal PCR Buffer (Takara), 0.2 ul dNTP (10 mM of each dNTP; Roche Diagnostics), and 0.25 ul of each primer (10 pmol ul ^ - 1^). All forward primers were labeled with tetrachloro-6-carboxy-fluorescein (TET). Reactions took place in a thermocycler (PTC-200, MJ Research) with an initial denaturation of 95°C for 5 minutes followed by 30 cycles of 95°C for 45 seconds, primer specific annealing temperature [57,58] for 30 seconds and 72°C for 45 seconds. Products were separated on 6% polyacrylamide gels and visualized with an FMBIO laser scanner (Hitachi). The base pair length of each allele was determined by running all samples with three marker individuals and a Genescan™-350 TAMRA size standard (Applied Biosystems). Analyses were conducted using FMBIO analysis software (Hitachi).

Microsatellite DNA genotyping is known to have a high occurrence of genotyping errors [60-62], and as such the following precautions were taken to minimize error during the scoring process. Firstly, scoring within and across gels was standardized. Since marker individuals were run multiple times, the most commonly computed value was taken as the true value for the base pair length of these individuals' alleles. Based on sequenced alleles, the computed alleles were shown to be quite accurate; within two base pairs of the true repeat length. Marker individuals, the TAMRA ladder and the slippage from incomplete PCR amplification were utilized to compute the base pair length of all other alleles. Microsatellite DNA 'slippage' or 'stutter bands' are common mistakes made during PCR amplification, where the *Taq *polymerase causes a mutation in the number of repeat motifs during the replication process [61]. Although these can be problematic for the scoring process, slippage can be used as a ladder, providing alleles are scored consistently. Secondly, six sets of four individuals (N = 24 of known genotype) were re-run to ensure that their alleles were scored correctly. The four individuals chosen were those that contained an allele scored as being the same base pair length and these were run beside each other on the same polyacrylamide gel. We tested whether allele j from individual X was the same as allele j from individual Y, Z and W at locus n. Alleles placed beside each other can easily be detected as being the same or different. When possible, secondary alleles were also re-scored to ensure that their predicted value, based on their relationships with other alleles on the same gel, was consistent with previous scoring efforts. Any incorrectly scored alleles were changed accordingly. Finally, MICRO-CHECKER version 1.0 [40] was used to test for the presence of scoring errors, large allele dropouts and/or null alleles. MICRO-CHECKER requires larger sample sizes to assess the latter and so two populations (sites 18 & 21) with the largest sample sizes (19 & 20) were used for these tests.

### Microsatellite DNA Data Analysis

The Bayesian model-based clustering programs STRUCTURE version 2.2 [41] and TESS version 2.1 [29] were employed to detect species level structure and assign individuals to groups. These methods use multi-locus genotypes and a predefined number of clusters (K) to generate groupings that minimize the deviation from Hardy-Weinberg equilibrium (HWE) and linkage equilibrium [29,55,56]. Individuals are then assigned probabilistically to one or more clusters based on their multi-locus genotype.

STRUCTURE implements a non-spatial, Bayesian clustering method that uses a Markov Chain Monte Carlo (MCMC) approach to explore the parameter space for the most likely estimates of the parameters Z ^(i) ^(individual i's population of origin), P (allele frequency in all populations) and α (admixture proportions for each individual; admixture model only) [55]. In contrast, TESS implements a spatial Bayesian clustering method using a Hidden Markov Random Field (HMRF) approach to model spatial dependency at the cluster membership level [56]. The HMRF concept accounts for the idea that individuals close together are more likely to be genetically similar with each other than individuals further apart and introduces a spatial dependency parameter ψ [29,56]. When ψ is set to zero the models used in TESS are virtually the same as STRUCTURE [29]. Therefore, TESS was used to determine whether the spatial location of the two clusters corresponded to the geographic areas of the two mtDNA clades identified by Jin *et al*. [39] and to assess the level of admixture between clusters.

STRUCTURE was used to identify the most likely number of clusters within the dataset (K _MAX_). Because Wang *et al*. identified strong population genetic structure in *P. vlangalii *of this area [48], we restricted the range of K from 1-4 as we were only interested in detecting 'species' level divergences. A total of 100 independent runs with 10,000-50,000 burn-in iterations and 100,000 post burn-in iterations were conducted at each value of K using default settings. This was a sufficient number of iterations to guarantee convergence. One hundred independent runs were completed to ensure that the multi-dimensional parameter space was sufficiently explored, removing the possibility of an MCMC chain getting stuck on one local optimum. Both an 'admixture' and 'no admixture' model were run because the 'admixture model' has a tendency to under-estimate the true value of K [63]. Thirteen independent runs with the highest LnP(D) were averaged and plotted using the statistical software package R [63,64]. This method for estimating K _MAX _is similar to that described for TESS [63]. Individual assignment probabilities, LnP(D) and convergence between runs were all used to assess the most likely value of K. In general, higher LnP(D) and large changes in the LnP(D) values between successive changes in the K parameter indicate a better fit to the data. Furthermore, a suitable K value should yield an individual assignment plot with a high probability of individual assignment. If greater than 80% of the individuals had a probability above 90% of being assigned to the identified clusters then the K parameter was considered to have sufficient power in explaining the data. Admixed individuals were those with less than 90% individual assignment probability and are expected to be rare.

Using the identified number of clusters from STRUCTURE, 100 independent iterations were run in TESS with individual spatial information. An admixture model was run with 50,000 burn-in iterations and 100,000 post burn-in iterations with ψ = 0.6 and α = 1.0. This was sufficient to ensure convergence within a single iteration. We ran 10 pilot runs and found that varying values of ψ = 0.6-2.0 and α = 1.0-6.0 did not change the overall assignment probabilities.

TESS requires unique individual coordinates to accurately depict spatial clustering. Since there were only site specific coordinates, each latitude and longitude were permutated slightly by a standard deviation of 0.015 using the "Generate Spatial Coordinates" function in TESS. This was necessary to more accurately depict individual clustering and its geographical association. CLUMPP version 1.1 [65] was used to average the 15 lowest DIC runs and to produce the admixture (Q) matrix. The level of admixture in each cluster was displayed graphically using the statistical package R [63].

### Mitochondrial DNA

DNA extractions from 146 individuals, that had a microsatellite DNA genotype, were selected for sequencing. Individuals that had a high assignment probability to one cluster or which showed evidence for an admixed genome were chosen. An 850 bp ND2 fragment was amplified in order to determine whether the clusters corresponded to the two mtDNA lineages outlined by Jin *et al*. [39]. Reactions took place in 25 ul volumes with 1 ul of DNA, 1 ul of ND2 forward and reverse primers (10 pmol ul ^- 1^) (L4447 5'-aag cag ttg ggc cca tgc ccc aaa aac gg- 3' and H5622- 5'- tat ttt aat taa aat atc tga gtt gca-3'; [66]), 2 ul dNTP (10 mM of each dNTP; Roche Diagnostics), 1.5 ul Mg ^2 + ^(25 mM), 2.5 ul 10× buffer (Takara), and 0.25 ul *Taq *polymerase (Takara). Thermal cycling was performed with an initial denaturation at 95°C for 5 minutes followed by 30 cycles of 95°C for 30 seconds, 50°C annealing for 30 seconds, 72°C for 45 seconds and a final extension of 72°C for 5 minutes. All PCR products were run on 1% agarose gels and purified using QIAquick PCR purification kits (Qiagen). The cleaned products were directly cycle sequenced in the forward direction using the L4447 primer. All DNA sequencing reactions were performed using BigDye terminator sequencing chemistry with an ABI 3730 sequencing machine (Applied Biosystems). Sequences were visualized and corrected using Sequencher version 4.5 (Genecode Corp) and aligned using MacClade version 4.08 [67].

A phylogenetic tree was generated to determine whether the data recovered the same clades as found by Jin *et al*. [39], and to assess whether the mtDNA lineages correspond to genotypic clusters found using the nuclear DNA. A Bayesian inference approach using MrBayes version 3.2 [42] was used. Akaike's Information Criterion (AIC) in MrModeltest version 2.1 [68] was used to determine the best-fit model. We used a "flat" prior and four Markov Chain Monte Carlo (MCMC) chains with 10,000,000 generations to ensure convergence. Tracer version 1.4 [69] was used to plot likelihood values and determine whether the Markov chains had reached convergence. Trees were sampled every 500 generations and the last 5,000 trees were used to estimate the consensus tree and Bayesian posterior probabilities. All prior trees were designated as "burn-in".

## Authors' contributions

DN collected and analyzed the data, and drafted the manuscript. JF conceived and designed the study, collected the samples and helped with data analysis and interpretation. YQ collected the samples and helped with drafting of the manuscript. All authors read and approved of the manuscript.
